# Increased prevalence of testicular microlithiasis in men with familial testicular cancer and their relatives

**DOI:** 10.1038/sj.bjc.6604704

**Published:** 2008-10-07

**Authors:** L A Korde, A Premkumar, C Mueller, P Rosenberg, C Soho, G Bratslavsky, M H Greene

**Affiliations:** 1 Clinical Genetics Branch, Division of Cancer Epidemiology and Genetics, National Cancer Institute, Rockville, MD, USA; 2 Diagnostic Radiology Department, National Institutes of Health Clinical Center, Bethesda, MD, USA; 3 Biostatistics Branch, Division of Cancer Epidemiology and Genetics, National Cancer Institute, Rockville, MD, USA; 4 Westat Inc, Rockville, MD, USA; 5 Urologic Oncology Branch, Center for Cancer Research, National Cancer Institute, Bethesda, MD, USA

**Keywords:** testicular microlithiasis, germ cell tumour, familial predisposition, genetic susceptibility, ultrasound, testicular cancer

## Abstract

Testicular germ cell tumours (TGCT) cluster in families, but responsible genes remain unidentified. The association between testicular microlithiasis (TM) and testicular carcinoma *in situ* (CIS) suggests that TM may be a TC risk factor. We report testicular ultrasound findings in men with familial TGCT (FTGCT) and their unaffected relatives. A total of 81 men (48 affected and 33 unaffected) from 31 families with ⩾2 TC cases underwent testicular ultrasound. Testicular microlithiasis was defined as either ‘classic’ (⩾5 microliths) or ‘limited’ (<5 microliths). Statistical analyses used Fisher's exact test and permutation testing. Testicular microlithiasis was more frequent in the contralateral testicles of men with a history of TGCT (affected men) than in unaffected men (48 *vs* 24%, *P*=0.04). The association appeared stronger for classic TM (21 *vs* 9%) than for limited TM (27 *vs* 15%). Testicular microlithiases were bilateral in six out of seven (87%) unaffected men. Among affected men, TM was not associated with histology, age at diagnosis or cancer treatment. Of the 31 families, 10 accounted for a majority (61%) of the TM cases identified (*P*=0.11). Testicular microlithiasis was more prevalent among FTGCT family members than described previously in the general population, and was more common among FTGCT cases *vs* unaffected blood relatives. Testicular microlithiasis appeared to cluster in certain families. These findings suggest both a familial predisposition to TM and an association between TM and FTGCT. If proven, this could be clinically important to men in FTGCT families, and may be useful in identifying specific genes involved in FTGCT.

Testicular germ cell tumours (TGCT) account for only 1% of malignancies in males, but constitute the most common cancer diagnosis among men aged 20–35 years (Edwards *et al*, 2005). A familial predisposition has been well documented: sons of men with TGCT consistently display a 4- to 6-fold increased risk of germ cell tumour compared with the general population, whereas brothers of affected siblings have an 8- to 10-fold increased risk (Dong and Hemminki, 2001; Hemminki and Li, 2004). However, specific susceptibility genes that predispose to testicular cancer have not yet been identified. Although an autosomal-recessive model provided the best data fit in the two segregation analyses performed to date (Nicholson and Harland, 1995; Heimdal *et al*, 1997), patterns of affection in multiple-case families are compatible with autosomal-dominant, autosomal-recessive and X-linked modes of inheritance, suggesting considerable genetic heterogeneity. Linkage analyses have revealed several potential genomic regions of interest, including 2p23, 3p12, 3q26, 12p13–q21, 18q21–q23 and Xq27 (Rapley *et al*, 2000; Crockford *et al*, 2006), and a specific deletion in the Y chromosome has also been identified as conferring an increased risk of both sporadic and familial testicular cancers in a small percentage of men (Nathanson *et al*, 2005). Overall, the preponderance of data suggest that no single major locus can account for the majority of the familial aggregation of TGCT, but rather that multiple susceptibility loci with weak effects appear to be involved (Crockford *et al*, 2006).

Although the specific aetiology of TGCT is not known, several additional risk factors have been described, including cryptorchid testes and contralateral testicular cancer (Garner *et al*, 2005). Skaakebeck has postulated the existence of a testicular dysgenesis syndrome, a constellation of findings including urogenital abnormalities, subfertility, testicular carcinoma *in situ* and, most importantly, TGCT. He proposed that this syndrome stems from abnormal gonadal development during embryogenesis and foetal life (Skakkebaek *et al*, 2001). Testicular microlithiasis (TM), often seen in association with carcinoma *in situ* (Holm *et al*, 2003), may be an additional manifestation of this disorder.

Testicular microlithiasis is characterised by the presence of calcium deposits within the seminiferous tubules. On ultrasound, these appear as 1–3 mm echogenic foci within the parenchyma of the testis. Prevalence estimates for TM in the general population range from 0.6 to 9% (Miller and Sidhu, 2002). The pathophysiology of these lesions is not well understood, but they are proposed to be caused by the degeneration of the seminiferous tubules (Dagash and Mackinnon, 2007). A number of medical conditions have been associated with TM, including cryptorchidism, varicocele, infertility and testicular torsion (Thomas *et al*, 2000; Zastrow *et al*, 2005). Importantly, numerous cross-sectional studies have shown an association between TM and testicular malignancy; in those studies, 8–54% of men with TM had concomitant TGCT (Ganem *et al*, 1999; Bach *et al*, 2001; Otite *et al*, 2001; Lam *et al*, 2007; Parenti *et al*, 2007). Although TM itself is not considered to be a problem requiring treatment, it is generally felt to be a marker of disease within the testes, and thus follow-up is often recommended.

One study (Coffey *et al*, 2007) has examined TM in the familial setting. Testicular germ cell tumour patients with and without a family history of TGCT, one unaffected male relative of each case and a group of healthy male controls underwent ultrasound examination. This study found a higher prevalence of TM among TGCT cases than controls, and a higher than expected prevalence of TM among male relatives of TGCT cases. This suggests a common genetic predisposition for TM and TGCT. In addition, TM could represent a biomarker of increased TGCT risk. In the context of familial disease, this could be particularly helpful in identifying individuals within a TGCT family who were at the highest risk of developing TGCT themselves.

Thorough evaluation of men with familial TGCT and their unaffected relatives may help to further define the familial testicular cancer syndrome phenotype, and might permit stratifying the population at familial risk into more homogenous, discrete clinical categories with similar genetic aetiologies, thereby improving the statistical power of gene discovery efforts. The National Cancer Institute's Clinical Genetics Branch (CGB) is conducting a multidisciplinary aetiologic study of familial testicular cancer aimed at ascertaining and studying families with TGCT. The study objectives are to (1) characterise the clinical phenotype of familial TGCT (Mai *et al*, 2007); (2) determine the underlying genetic mechanism(s) for susceptibility to TGCT in families (Rapley *et al*, 2000; Nathanson *et al*, 2005; Crockford *et al*, 2006; Coffey *et al*, 2007; Mueller *et al*, 2007); (3) evaluate psychosocial and behavioural issues resulting from being a member of a family at increased risk of TGCT (Peters *et al*, 2006, 2008) and (4) create a repository of annotated biospecimens to permit translational research investigations. In this analysis, we present the results of testicular ultrasound (TUS) examinations performed during a comprehensive clinical evaluation in a subset of families enrolled on the CGB FTGCT study.

## Materials and methods

Families with two or more cases of documented GCT in blood relatives (at least one of which was testicular in origin) or a single family member with bilateral testicular cancer were eligible for the FTGCT study. An eligible family was defined as having ⩾2 objectively confirmed testicular or extragonadal GCTs. First-degree relatives of a case who were ⩾12 years and spouses of a case who had children participating were invited to enroll in the study. In addition, non-first-degree blood relatives who provided a genetic link between two cases, and blood relatives with cancer other than GCT were invited to participate.

Participants completed detailed family history, medical history, risk factor and psychosocial/behavioural questionnaires, and were asked to provide a research blood sample. For families in which there were two or more affected males, the affected individuals, their spouses and first-degree relatives were invited to travel to the National Institutes of Health (NIH) Clinical Center for a thorough diagnostic and research evaluation, including a detailed physician history and physical examination, laboratory testing, semen analysis (males aged ⩾18 years with at least one remaining testicle), ultrasound imaging of the testes or ovaries and computed tomography or ultrasound of the kidneys. All participants also attended a genetic counselling and education session. This study has been reviewed and approved by the NCI Institutional Review Board (NCI Protocol 02-C-0178), and all participants provided written informed consent.

Ultrasound studies were performed at the NIH Clinical Center Diagnostic Radiology Department between January 2003 and November 2005 on one of two machines: the Acuson Sequoia 512 with a 14 MHz probe or the Phillips HDI 5000 SonoCT with a 12 MHz probe. All ultrasound examinations were reviewed by a single radiologist (AP). We compared specific ultrasonographic findings between the affected and unaffected individuals, and analysed the data for associations between TM and specific histological findings, as well as pertinent medical and surgical history. We also investigated the possibility of familial TM clustering by analysing the prevalence of TM in participating families. We defined testicular microcalcification as either ‘classic’ TM (⩾5 microliths) or ‘limited’ TM (<5 microliths), as the latter has also been associated with an increased risk of TC (Dagash and Mackinnon, 2007). Statistical analyses were performed using SAS (version 9.13). *P*-values for prevalence rates were calculated using Fisher's exact test. We assessed clustering of microlithiasis within families using a permutation test to account for the sparse numbers of affected men within families and men at risk, respectively. The permutation test randomly shuffled family indicators across subjects and testes indicators across men, respectively, and used the Pearson *χ*^2^ as a measure of departure from homogeneity. *P*-values were computed from 10 000 random shuffles.

## Results

We have enrolled 506 members (including 140 cases) of 99 families. This report focuses on the TUS examinations performed on 48 affected males and 33 unaffected male blood relatives from 31 multiple-case testicular cancer families who elected to come to NIH. Of these 31 families, 14 had two affected brothers, 6 consisted of affected father–son pairs, three contained two affected cousins and 8 had more than two affected family members (see Table 1). All unaffected men were first-degree relatives of cases (affected father=10, affected son=8, affected brother=11 and affected father and brother=4). Cases had unilateral ultrasound performed on their remaining testicle, whereas unaffected family members had both testes examined, with two exceptions: one affected male had a pineal germ cell tumour, and therefore underwent bilateral TUS, and one unaffected male had a previous unilateral orchiectomy to treat cryptorchidism in the context of his family history of TGCT. Furthermore, one male with bilateral testicular cancer who had a partial orchiectomy at the time of his second TC diagnosis is included.

The mean age of study participants was 39 years. The mean age at TC diagnosis was 31.5 years. One incident case of TGCT has been detected during 5 years of prospective study follow-up (see below for additional details). Four patients had focal testicular lesions for which further workup was recommended. On repeat examination, three were thought to be intratesticular cysts. One patient with a 3-mm hypoechoic density was followed with periodic ultrasounds for 2 years, without change; no further evaluation was performed.

The prevalence of urogenital abnormalities among study participants is shown in Table 2. No statistically significant differences were noted in the prevalence of hydrocele or varicocele. Epididymal cysts were more common in unaffected men (61 *vs* 40%, *P*=0.06). Of the 20 unaffected men with epididymal cysts, 65% had unilateral cysts and 35% had bilateral findings. Overall, epididymal cysts were seen in 41% (46 out of 113) of testes imaged.

**Figure 1:**
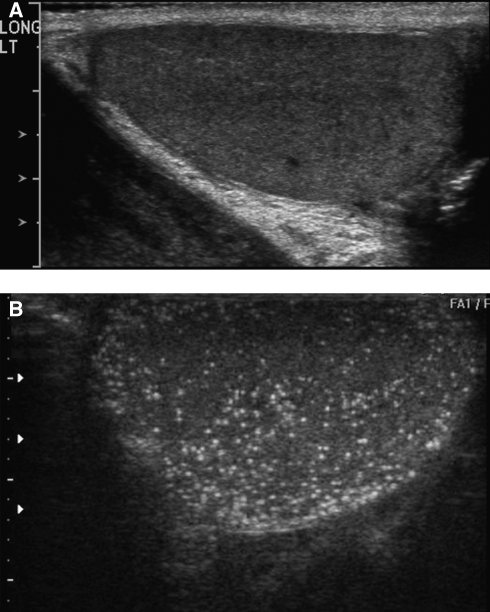
Testicular ultrasounds depicting (**A**) normal exam and (**B**) extensive microlithiasis.

Testicular microlithiasis (classic plus limited) occurred more frequently in the contralateral testes of cases than in unaffected men (48 *vs* 24%, *P*=0.04). Among the unaffected men with calcifications, the TMs were bilateral in six out of seven (87%). Figure 1A shows a representative normal TUS and an ultrasound of a male with extensive microlithiasis is shown in Figure 1B.

Table 3 lists additional factors that are potentially associated with TM. Five out of six men who reported infertility at the time of ultrasound examination had TM. As expected, cryptorchidism was more frequent among cases than among unaffected men (12 *vs* 3%); however, the presence of TM did not correlate with history of cryptorchidism in the full cohort of men studied. The rate of previous inguinal hernia was similar among cases and unaffecteds (26 *vs* 18%), and was not correlated with TM. Similarly, no associations were noted between TM and history of infection (orchitis or epididymitis), testicular injury or specific cancer treatments. The incidence of TM did neither vary by age at ultrasound nor by age at diagnosis among men with a history of testicular cancer (data not shown). The prevalence of TM among affected males did not differ by TC histology.

In this study, 8 out of 31 (26%) families had ⩾3 family members with a history of TGCT and 23 out of 31 had two cases. Testicular microlithiasis was more common among families with two affected family members than in those with multiple affected family members (65 *vs* 35%), but this result was not statistically significant.

We also looked for evidence of family clustering of microlithiasis. In 27 out of 31 families included in this study, ultrasound exams were performed on ⩾2 family members (total of 76 participants); in the remaining four families only one family member was examined. In 12 out of 27 families (44%), there were no cases of TM (31 family members screened); 5 out of 27 (19%) families showed TM in <50% (5 out of 16 persons) of family members screened, and the remaining 10 out of 27 (37%) families had TM in ⩾50% (19 out of 49 persons) of family members screened. These latter 10 families accounted for 61% of TM cases identified in this study. The permutation test *P*-value approached statistical significance (*P*=0.11), giving some evidence for familial clustering.

**Figure 2:**
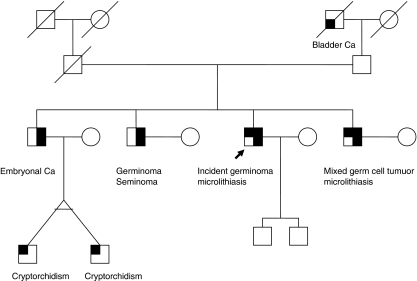
Pedigree of a family in which multiple members have testicular cancer and microlithiasis.

During 5 years of study follow-up, one incident case of TGCT has been diagnosed. This patient had bilateral classic TM at the time of his study ultrasound in November 2005, and periodic follow-up ultrasonography was recommended. A repeat ultrasound in September 2007 showed stable findings. The patient noticed a testicular lump on self-examination in January 2008, which led to a repeat ultrasound and subsequent diagnosis of TGCT. Retrospective review of his previous imaging studies revealed no evidence of testicular mass. It can be noted that this patient is one of four brothers; two of his brothers had a previous diagnosis of unilateral testicular cancer (one had microlithiasis in his contralateral testicle at the time of his NIH evaluation and the other did not) and one had bilateral testicular cancer, all before enrolling in the study. The family pedigree is shown in Figure 2.

## Discussion

We found a striking 48% prevalence of TM among men with a history of TGCT, and a higher than expected prevalence (24%) in their unaffected male family members. In contrast, studies in the general population have shown much lower rates of TM, with prevalence estimates ranging from 0.6 to 9% (Peterson *et al*, 2001; Miller and Sidhu, 2002; Serter *et al*, 2006). In affected men, we found no association between TM and the type of treatment or TGCT histology. In addition, our data indicate that TM may cluster in certain families. These findings raise the possibility that TM may be an inherited condition that predisposes to testicular cancer among men in FTGCT kindred.

It is also notable that the one incident cancer case in this study occurred in a male from a family with both microlithiasis and a rather remarkable family history, with four affected brothers, one of whom had bilateral disease. To our knowledge, this is the first report of an incident TGCT occurring during prospective follow-up of a multiple-case family. It has been recently suggested that all men with bilateral TGCT share a hereditary predisposition (Harland *et al*, 2007); our findings lend additional credence to this hypothesis. Thus, the combination of a significant family history and the finding of TM on ultrasound may identify a population of men that warrant close follow-up for the development of TGCT.

These data support an association between TM and risk of FTGCT. In studies of symptomatic men who underwent ultrasound evaluation, the relative risk of TGCT in men found to have microlithiasis, as compared with those without the condition, ranged from 21.6 to 36.5 (Cast *et al*, 2000; Ringdahl *et al*, 2004). We observed both a high prevalence of contralateral TM in men with a history of unilateral TGCT and a greater than expected prevalence of TM in unaffected male relatives.

Another study has examined the association between TM and TGCT in the familial context. Coffey *et al* recently reported the results of an analysis that included TGCT cases with and without a family history of TGCT, male family members and normal male controls. They found a greater prevalence of TM among cases than among controls (43.9 *vs* 17.8%) and noted a similarly high prevalence of TM among family members of cases with a family history of TGCT (45.5%). The prevalence rates seen in that study were remarkably similar to those presented here, and substantially higher than those reported in the general population (Peterson *et al*, 2001; Miller and Sidhu, 2002; Serter *et al*, 2006). Our findings provide further evidence of an association between TM and TGCT, specifically among individuals in FTGCT families. If confirmed, these data could have important implications for screening in these high-risk families by identifying a subset of family members who might be at particularly elevated TGCT risk.

In addition, this finding may be important in the context of efforts aimed at identifying susceptibility genes for testicular cancer. By designating at-risk family members with TM as ‘affected’ in linkage analyses, it may be possible to significantly increase the statistical power of gene-finding studies, which are currently constrained, in significant part, by the limited number of affected men in multiple-case families.

Alternative explanations for the high prevalence of TM among affected men in our study include a shared association with other known TGCT risk factors, and late effects of cancer-related chemotherapy and/or radiation therapy. Previous studies have suggested that TM is more frequent among men with infertility and those with cryptorchidism (Thomas *et al*, 2000; Zastrow *et al*, 2005), both of which are established TGCT risk factors. In our study, only six men reported infertility, all of whom had TGCT and all but one had TM. As this was a retrospective analysis, we cannot determine whether men reporting infertility had this condition before their diagnosis of TGCT or whether it was treatment-related. Only seven men in our study had a history of cryptorchidism; three of whom also had TM. Given the small numbers in our study, it is impossible to rule out a contribution of these factors to our finding of a high prevalence of TM. In reviewing treatment history, neither chemotherapy nor radiation therapy was associated with an increased risk of TM in our cases. In addition, the high prevalence of TM among unaffected men makes a treatment effect unlikely.

Lastly, our data suggest that TM may cluster in certain families. In our study, less than half of the families accounted for a majority of the cases of TM, whereas another large subset of families had no TM in any family members. Coffey *et al* noted a higher degree of concordance for TM among TGCT cases and matched relative pairs than was expected by chance (Coffey *et al*, 2007). This study supports and, in fact, strengthens this observation, as we were able to examine multiple individuals in many of the families we studied. Taken together, these results suggest the presence of a genetic susceptibility to TM, which may also confer an increased risk of TGCT.

Although provocative, the findings in our study are limited by several factors. The small sample size and cross-sectional study design preclude drawing directional or causal conclusions. The study population is a convenience sample of volunteers; upon enrollment in the study, families were asked about their willingness to travel to the NIH Clinical Center for an in-person evaluation. Those included in the current analysis may not be truly representative of the entire population of FTGCT families. Finally, we did not have a non-familial control population, and therefore relied upon the literature for estimates of TM prevalence in the general population. However, this unique population of men with a familial predisposition to testicular cancer has not been extensively studied, and thus our findings contribute significantly to the understanding of familial testicular cancer. These novel, although exploratory, observations will serve as the basis for larger, more carefully designed studies that might prove definitive.

A particular strength of this study is that all participants underwent a comprehensive clinical evaluation by a genetics specialty team, and systematically provided detailed medical and family history information. In addition, all ultrasound films were read by a single radiologist for consistency, and meticulous attempts were made to obtain comparison and/or follow-up studies on patients with abnormalities discovered. Furthermore, we were able to study multiple affected and unaffected men in our families, thus strengthening the observation that TM appears to cluster in particular families. We are currently in the process of bringing additional family members to the Clinical Center for ultrasound screening, to further investigate the association between TGCT and TM, and to evaluate the hypothesis that TM may be a useful marker of risk in men from FTGCT kindreds.

This study is among the first to examine TUS findings among men with FTGCT and also the first to report an incident TGCT in an at-risk family member during prospective follow-up. Our finding of an unusually high prevalence of TM among men with familial TGCT and their unaffected family members is in keeping with that previously reported by Coffey *et al*. Our study further suggests that TM clusters in certain families, a finding that we are hoping to confirm by imaging additional members of our FTGCT families. We propose that there may be a genetic predisposition to TM, which is on the causal pathway to TGCT. If this can be proven, we will be a significant step closer to identifying the specific genes that confer susceptibility to TGCTs.

## Figures and Tables

**Table 1:** Family relationships and the number of affected and unaffected family members included in analysis

**Family**	**Relationship among affected family members**	**No. of affected**	**No. of unaffected**
1	Siblings	0	2
2	>2 affected family members	1	1
3	>2 affected family members	2	1
4	Father/son	1	1
5	>2 affected family members	2	1
6	Siblings	2	1
7	Siblings	2	1
8	Cousins (mixed maternal/paternal)	2	1
9	Siblings	2	1
10	Father/son	2	0
11	Cousins (maternal)	2	2
12	Siblings	1	0
13	>2 affected family members	2	2
14	>2 affected family members	2	3
15	Father/son	2	2
16	Father/son	1	2
17	Siblings	1	0
18	Siblings	2	1
19	Cousins (paternal)	1	1
20	Siblings	1	0
21	Siblings	1	1
22	>2 affected family members	2	1
23	Siblings	1	2
24	>2 affected family members	1	2
25	Siblings	2	2
26	Father/son	2	0
27	>2 affected family members	2	1
28	Father/son	2	0
29	Siblings	1	0
30	Siblings	2	1
31	Siblings	1	1

**Table 2:** Prevalence of ultrasound findings among affected and unaffected men in the CGB familial testicular cancer study

**Condition**	**Prevalence (95% CI) in unaffecteds**	**Prevalence (95% CI) in affecteds**	***P*-value**
Varicocele	5/33 (15%)	10/48 (21%)	0.57
	(5–32)	(10–35)	
			
Hydrocele	4/33 (12%)	6/48 (13%)	1.00
	(3–28)	(5–25)	
			
Epididymal cyst	20/33 (61%)	19/48 (40%)	0.06
	(42–77)	(26–55)	
			
**Microcalcifications**	**8/33** (**24%)**	**23/48** (**48%)**	**0.04**
	**(11–42)**	**(33–63)**	

Bold values indicate statistically significant findings.

^*^P-value for comparison of the degree of TM among affected *vs* unaffected study participants.

**Table 3:** Associations between TM and other conditions in FTC families

**Condition**	**TM**	**No TM**
Infertility (*n*=6)^†^	5/6	1/6
Inguinal hernia (*n*=18)	8/18	10/18
Cryptorchidism (*n*=7)	3/7	4/7
Orchitis/epididymitis (*n*=5)	3/5	2/5
STD (*n*=9)	5/9	4/9
Testicular injury (*n*=7)	2/7	5/7
		
*Treatment* a		
History of chemotherapy (*n*=18)	10/18	8/18
History of radiation therapy (*n*=21)	9/21	12/21
		
*Histology* a		
Seminoma (*n*=23)	10/23	13/23
Non-seminoma (*n*=23)	11/23	12/23
Embryonal carcinoma	3/6	3/6
Mixed GCT	5/11	6/11
Malignant teratoma	3/5	2/5
Yolk sac/endodermal sinus tumor	0/1	1/1

^†^*P*=0.06.

a Among affected men only (*n*=46; two patients had histology listed in the pathology report as germinoma, NOS).

## References

[bib1] Bach AM, Hann LE, Hadar O, Shi W, Yoo HH, Giess CS, Sheinfeld J, Thaler H (2001) Testicular microlithiasis: what is its association with testicular cancer? Radiology 220: 70–75 11425975 10.1148/radiology.220.1.r01jl3670

[bib2] Cast JE, Nelson WM, Early AS, Biyani S, Cooksey G, Warnock NG, Breen DJ (2000) Testicular microlithiasis: prevalence and tumor risk in a population referred for scrotal sonography. AJR Am J Roentgenol 175: 1703–1706 11090407 10.2214/ajr.175.6.1751703

[bib3] Coffey J, Huddart RA, Elliott F, Sohaib SA, Parker E, Dudakia D, Pugh JL, Easton DF, Bishop DT, Stratton MR, Rapley EA (2007) Testicular microlithiasis as a familial risk factor for testicular germ cell tumour. Br J Cancer 97: 1701–1706 17971766 10.1038/sj.bjc.6604060 PMC2360292

[bib4] Crockford GP, Linger R, Hockley S, Dudakia D, Johnson L, Huddart R, Tucker K, Friedlander M, Phillips KA, Hogg D, Jewett MA, Lohynska R, Daugaard G, Richard S, Chompret A, Bonaiti-Pellie C, Heidenreich A, Albers P, Olah E, Geczi L, Bodrogi I, Ormiston WJ, Daly PA, Guilford P, Fossa SD, Heimdal K, Tjulandin SA, Liubchenko L, Stoll H, Weber W, Forman D, Oliver T, Einhorn L, McMaster M, Kramer J, Greene MH, Weber BL, Nathanson KL, Cortessis V, Easton DF, Bishop DT, Stratton MR, Rapley EA (2006) Genome-wide linkage screen for testicular germ cell tumour susceptibility loci. Hum Mol Genet 15: 443–451 16407372 10.1093/hmg/ddi459

[bib5] Dagash H, Mackinnon EA (2007) Testicular microlithiasis: what does it mean clinically? BJU Int 99: 157–160 17026598 10.1111/j.1464-410X.2006.06546.x

[bib6] Dong C, Hemminki K (2001) Modification of cancer risks in offspring by sibling and parental cancers from 2,112,616 nuclear families. Int J Cancer 92: 144–150 11279618

[bib7] Edwards BK, Brown ML, Wingo PA, Howe HL, Ward E, Ries LA, Schrag D, Jamison PM, Jemal A, Wu XC, Friedman C, Harlan L, Warren J, Anderson RN, Pickle LW (2005) Annual report to the nation on the status of cancer, 1975–2002, featuring population-based trends in cancer treatment. J Natl Cancer Inst 97: 1407–1427 16204691 10.1093/jnci/dji289

[bib8] Ganem JP, Workman KR, Shaban SF (1999) Testicular microlithiasis is associated with testicular pathology. Urology 53: 209–213 9886614 10.1016/s0090-4295(98)00438-5

[bib9] Garner MJ, Turner MC, Ghadirian P, Krewski D (2005) Epidemiology of testicular cancer: an overview. Int J Cancer 116: 331–339 15818625 10.1002/ijc.21032

[bib10] Harland SJ, Rapley EA, Nicholson PW (2007) Do all patients with bilateral testis cancer have a hereditary predisposition? Int J Androl 30: 251–255 17705806 10.1111/j.1365-2605.2007.00801.x

[bib11] Heimdal K, Olsson H, Tretli S, Fossa SD, Borresen AL, Bishop DT (1997) A segregation analysis of testicular cancer based on Norwegian and Swedish families. Br J Cancer 75: 1084–1087 9083348 10.1038/bjc.1997.185 PMC2222754

[bib12] Hemminki K, Li X (2004) Familial risk in testicular cancer as a clue to a heritable and environmental aetiology. Br J Cancer 90: 1765–1770 15208620 10.1038/sj.bjc.6601714 PMC2410275

[bib13] Holm M, Hoei-Hansen CE, Rajpert-De Meyts E, Skakkebaek NE (2003) Increased risk of carcinoma *in situ* in patients with testicular germ cell cancer with ultrasonic microlithiasis in the contralateral testicle. J Urol 170: 1163–1167 14501716 10.1097/01.ju.0000087820.94991.21

[bib14] Lam DL, Gerscovich EO, Kuo MC, McGahan JP (2007) Testicular microlithiasis: our experience of 10 years. J Ultrasound Med 26: 867–873 17592049 10.7863/jum.2007.26.7.867

[bib15] Mai PL, Korde L, Kramer J, Peters J, Mueller CM, Pfeiffer S, Stratakis CA, Pinto PA, Bratslavsky G, Merino M, Choyke P, Linehan WM, Greene MH (2007) A possible new syndrome with growth-hormone secreting pituitary adenoma, colonic polyposis, lipomatosis, lentigines and renal carcinoma in association with familial testicular germ cell malignancy: a case report. J Med Case Reports 1: 9 10.1186/1752-1947-1-9 PMC1847830 17411461

[bib16] Miller FN, Sidhu PS (2002) Does testicular microlithiasis matter? A review. Clin Radiol 57: 883–890 12413911 10.1053/crad.2002.1005

[bib17] Mueller CM, Korde L, Katki HA, Rosenberg PS, Peters JA, Greene MH (2007) Constitutional cytogenetic analysis in men with hereditary testicular germ cell tumor: no evidence of disease-related abnormalities. Cancer Epidemiol Biomarkers Prev 16: 2791–2794 18086791 10.1158/1055-9965.EPI-07-0521 PMC3125977

[bib18] Nathanson KL, Kanetsky PA, Hawes R, Vaughn DJ, Letrero R, Tucker K, Friedlander M, Phillips KA, Hogg D, Jewett MA, Lohynska R, Daugaard G, Richard S, Chompret A, Bonaiti-Pellie C, Heidenreich A, Olah E, Geczi L, Bodrogi I, Ormiston WJ, Daly PA, Oosterhuis JW, Gillis AJ, Looijenga LH, Guilford P, Fossa SD, Heimdal K, Tjulandin SA, Liubchenko L, Stoll H, Weber W, Rudd M, Huddart R, Crockford GP, Forman D, Oliver DT, Einhorn L, Weber BL, Kramer J, McMaster M, Greene MH, Pike M, Cortessis V, Chen C, Schwartz SM, Bishop DT, Easton DF, Stratton MR, Rapley EA (2005) The Y deletion gr/gr and susceptibility to testicular germ cell tumor. Am J Hum Genet 77: 1034–1043 16380914 10.1086/498455 PMC1285161

[bib19] Nicholson PW, Harland SJ (1995) Inheritance and testicular cancer. Br J Cancer 71: 421–426 7841065 10.1038/bjc.1995.86 PMC2033581

[bib20] Otite U, Webb JA, Oliver RT, Badenoch DF, Nargund VH (2001) Testicular microlithiasis: is it a benign condition with malignant potential? Eur Urol 40: 538–542 11752862 10.1159/000049832

[bib21] Parenti GC, Zago S, Lusa M, Campioni P, Mannella P (2007) Association between testicular microlithiasis and primary malignancy of the testis: our experience and review of the literature. Radiol Med (Torino) 112: 588–596 17563846 10.1007/s11547-007-0165-1

[bib22] Peters JA, Beckjord EB, Banda Ryan DR, Carr AG, Vadaparampil ST, Loud JT, Korde L, Greene MH (2008) Testicular cancer and genetics knowledge among familial testicular cancer family members. J Genet Couns 17: 351–364 18481162 10.1007/s10897-008-9153-4 PMC3111072

[bib23] Peters JA, Vadaparampil ST, Kramer J, Moser RP, Court LJ, Loud J, Greene MH (2006) Familial testicular cancer: interest in genetic testing among high-risk family members. Genet Med 8: 760–770 17172939 10.1097/01.gim.0000250506.15979.0c

[bib24] Peterson AC, Bauman JM, Light DE, McMann LP, Costabile RA (2001) The prevalence of testicular microlithiasis in an asymptomatic population of men 18 to 35 years old. J Urol 166: 2061–2064 11696707

[bib25] Rapley EA, Crockford GP, Teare D, Biggs P, Seal S, Barfoot R, Edwards S, Hamoudi R, Heimdal K, Fossa SD, Tucker K, Donald J, Collins F, Friedlander M, Hogg D, Goss P, Heidenreich A, Ormiston W, Daly PA, Forman D, Oliver TD, Leahy M, Huddart R, Cooper CS, Bodmer JG, Easton DF, Stratton MR, Bishop DT (2000) Localization to Xq27 of a susceptibility gene for testicular germ-cell tumours. Nat Genet 24: 197–200 10655070 10.1038/72877

[bib26] Ringdahl E, Claybrook K, Teague JL, Northrup M (2004) Testicular microlithiasis and its relation to testicular cancer on ultrasound findings of symptomatic men. J Urol 172: 1904–1906 15540751 10.1097/01.ju.0000142449.47356.29

[bib27] Serter S, Gumus B, Unlu M, Tuncyurek O, Tarhan S, Ayyildiz V, Pabuscu Y (2006) Prevalence of testicular microlithiasis in an asymptomatic population. Scand J Urol Nephrol 40: 212–214 16809262 10.1080/00365590600589641

[bib28] Skakkebaek NE, Rajpert-De Meyts E, Main KM (2001) Testicular dysgenesis syndrome: an increasingly common developmental disorder with environmental aspects. Hum Reprod 16: 972–978 11331648 10.1093/humrep/16.5.972

[bib29] Thomas K, Wood SJ, Thompson AJ, Pilling D, Lewis-Jones DI (2000) The incidence and significance of testicular microlithiasis in a subfertile population. Br J Radiol 73: 494–497 10884745 10.1259/bjr.73.869.10884745

[bib30] Zastrow S, Hakenberg OW, Wirth MP (2005) Significance of testicular microlithiasis. Urol Int 75: 3–7 16037699 10.1159/000085918

